# Genomic characterization of a pathogenic *Bacillus licheniformis* strain LSDY01: deciphering its genetic diversity and virulence-associated traits

**DOI:** 10.3389/fmicb.2026.1815181

**Published:** 2026-06-02

**Authors:** Lingfeng Liang, Zhenqiu Shang, Aixia Liu, De Lin, Ningjun Wu, Jin Jing, Ziqi Yang, Wugao Liu

**Affiliations:** 1 Department of Infectious Diseases, Lishui Hospital of Wenzhou Medical University, The First Affiliated Hospital of Lishui University, Lishui People's Hospital, Lishui, Zhejiang, China; 2 Department of Clinical Laboratory, Lishui Hospital of Wenzhou Medical University, The First Affiliated Hospital of Lishui University, Lishui People's Hospital, Lishui, Zhejiang, China; 3 Guangxi Medical University Oncology School, Nanning, China

**Keywords:** *Bacillus licheniformis*, horizontal gene transfer, infection, T4SS, virulence factors

## Abstract

**Background:**

*Bacillus licheniformis* is an opportunistic pathogen in clinical settings. However, the emergence of clinical strains carrying horizontally acquired virulence determinants, including chromosomal genomic islands harboring *yopX*, a putative type IV secretion system (T4SS), and plasmids bearing toxin-antitoxin systems and additional virulence factors, poses a significant challenge to diagnosis and treatment. Moreover, the genetic basis of the pathogenicity of clinical isolates has not been comprehensively studied.

**Methods:**

A pathogenic *B. licheniformis* strain (LSDY01) isolated from a skin infection was subjected to whole-genome sequencing and comparative genomic analyses. Phylogenetic reconstruction, pan-genome analysis, and detailed characterization of plasmid and chromosomal virulence determinants were performed. Antimicrobial susceptibility testing was performed according to standardized guidelines. Biofilm formation assays were also conducted. The cytotoxic effect of LSDY01 on HEK293 cells was evaluated using a CCK-8 assay.

**Results:**

Strain LSDY01 belonged to *B. licheniformis* ST20, differing by only one allele from the prevalent ST3. Its closest relatives were the Daqu-derived strains CP143961.1 and CP143962.1. A unique horizontally acquired genomic island (~157 27 kb, GC 33.03%) and a putative type IV secretion system (T4SS) gene cluster were identified on the chromosome of this strain. A novel plasmid (pLSDY01), which is highly similar to environmental plasmids, harbors *yopX*, a toxin-antitoxin system, *pilT*, and a pistol ribozyme. LSDY01 was susceptible to imipenem and vancomycin but resistant to penicillin, erythromycin, and chloramphenicol. The CCK-8 assay revealed a non-significant trend toward reduced HEK293 cell viability after co-culture with LSDY01 (*p* = 0.0545 at 2 h of CCK-8 incubation).

**Conclusion:**

Our findings suggest that horizontal gene transfer, including plasmid acquisition and potential phage integration, may have enabled *B. licheniformis* to evolve into a pathogen, highlighting the need to reassess the safety of traditionally non-pathogenic microbes.

## Highlights

- First report on the genetic diversity and virulence traits of pathogenic *Bacillus licheniformis.*
- LSDY01 carries a unique virulence arsenal acquired via horizontal gene transfer, including a 157-kb genomic island, T4SS, and a novel plasmid harboring *yopX* and *pilT*.
- The plasmid showed high homology to environmental plasmids, suggesting cross-environmental dissemination of virulence determinants.
- This study suggests how horizontal gene transfer drives the evolution of *Bacillus licheniformis* from a commensal microbe to an opportunistic pathogen.

## 1 Introduction

*Bacillus licheniformis* is a Gram-positive, spore-forming bacterium widely utilized in industry but increasingly recognized as an opportunistic pathogen (Muras et al., 2021; Shleeva et al., 2023). Reports link it to bacteremia, endocarditis, and soft tissue infections, particularly in immunocompromised hosts, underscoring the need to decipher its pathogenic determinants (Zou et al., 2024; Deniz et al., 2025). Bacterial pathogenicity is often enabled by mobile genetic elements (MGEs), such as plasmids and genomic islands, and temperate bacteriophages (prophages), which harbor virulence and fitness genes (Gal-Mor and Finlay, 2006; Boyd and Brüssow, 2002). Through lysogenic conversion, phages can introduce key virulence factors, such as toxins, superantigens, and effector proteins directly into the bacterial genome, thereby converting benign strains into pathogenic variants (Brüssow et al., 2004; Kobakhidze et al., 2024). These elements facilitate the horizontal acquisition of traits, including biofilm formation, antibiotic resistance, and siderophore systems, which are critical for host adaptation and immune evasion (Mazzamurro et al., 2025; Weisberg and Chang, 2023). In particular, phage-mediated gene transfer plays a crucial role in the rapid evolution of bacterial pathogens, enabling them to acquire novel virulence mechanisms and adapt to new niches. However, the specific genetic mechanisms driving the virulence of clinical *B. licheniformis* isolates remain poorly understood. It remains unclear whether prophage-encoded virulence factors contribute to the pathogenicity of these strains. This study identified a clinical *B. licheniformis* strain, LSDY01, carrying a novel plasmid harboring a toxin-antitoxin system and *yopX*, along with a horizontally acquired chromosomal genomic island containing an anti-CRISPR gene (*AcrIIA7*) and *yopX*. Notably, a functional type IV secretion system (T4SS) was also identified on the chromosome, a rare feature in this species, which may enhance virulence through effector delivery. This study delineates a distinct virulent lineage of *B. licheniformis* and establishes a genetic framework for understanding its transition from commensal to opportunistic pathogen.

## 2 Materials and methods

### 2.1 Bacterial strain

**Figure 1:**
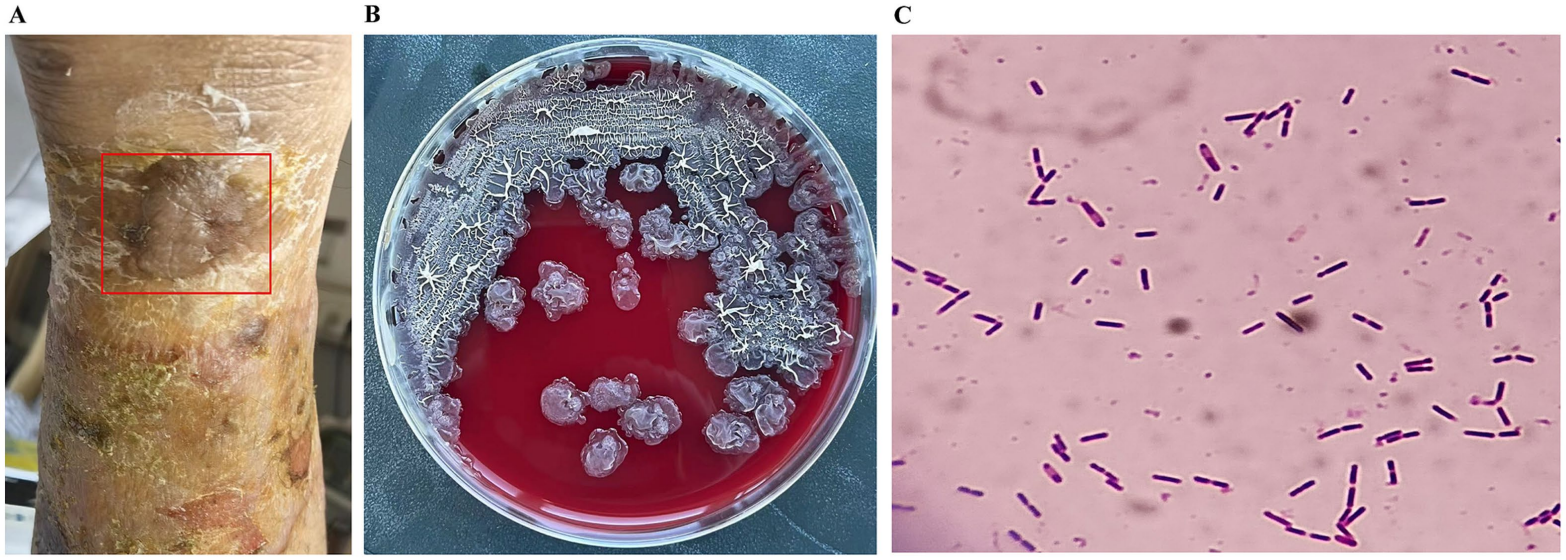
Clinical presentation, culture characteristics, and Gram stain of *Bacillus* species from a lower leg blister infection. **(A)** Blister lesion on the lower leg of the patient. **(B)** Bacterial growth on Columbia blood agar after 48 h of incubation, showing typical morphology of *Bacillus* spp. **(C)** Gram stain of the wound exudate revealing Gram-positive bacilli (scale bar: 10 μm).

LSDY01 was isolated from a 42-year-old male patient in a tertiary teaching hospital (Lishui, China) in July 2024. The patient’s right lower limb was erythematous and swollen for a week (Figure 1A). Despite a 4-day course of outpatient ceftriaxone therapy, his condition did not improve. After culturing the wound exudate on Columbia blood agar plate (Antu, China) at 35 °C and 5% CO2 atmosphere for 2 days, a dry, rough, wrinkled (lichenoid), and spreading appearance non-hemolytic Gram-positive bacilli was obtained and named LSDY01 (Figures 1B,C) and subsequent matrix-assisted laser desorption time-of-flight mass spectrometry (MALDI-TOF MS) (Bruker Daltonik GmbH, Bremen, Germany) definitively identified the pathogen as *B. licheniformis*. Antimicrobial susceptibility testing was then performed using the broth microdilution method according to the Clinical and Laboratory Standards Institute (CLSI) guidelines document M45 (Edition 2016), and the results were interpreted based on the criteria provided therein (CLSI, 2015). The isolate exhibited susceptible to imipenem, vancomycin, amikacin, and gentamicin, but remained resistant to penicillin, ampicillin, erythromycin, clindamycin, trimethoprim-sulfamethoxazole, and chloramphenicol; consistent with its intrinsic resistance to ceftriaxone. The antibiotic therapy was then escalated to vancomycin, targeting the confirmed pathogen. The patient’s clinical condition improved steadily, and he was discharged after recovery.

### 2.2 Whole-genome sequencing and bioinformatic analysis

LSDY01 was cultured in Luria-Bertani (LB) broth at 37 °C for 16 h. The genomic DNA was extracted using an AxyPrep bacterial genomic DNA miniprep kit (Axygen Scientific, Union City, CA, USA) according to the manufacturer’s instructions. Whole-genome sequencing (WGS) was conducted using the NovaSeq 6000 (Illumina, California, USA) and Nanopore (Nanopore, Oxford, UK) platforms at Novogene Bioinformatics Technology Co., Ltd. (Tianjin, China). The derived short reads and long reads were assembled by using Unicycler software (v0.5.0). The genome sequences were annotated using Bakta v1.11.3 (Beyvers et al., 2025), and then corrected using BLAST searches against the UniProtKB/Swiss-Prot and RefSeq databases. The clusters of orthologous groups (COG) classification of the predicted genes were achieved by eggNOG-mapper v2 (Cantalapiedra et al., 2021). Genome-based taxonomy classification was conducted using Type Strain Genome Server (TYGS) tools (Meier-Kolthoff and Göker, 2019). Genome sequences were compared using FastANI (v1.33) to determine pairwise Average Nucleotide Identity (Jain et al., 2018). A species boundary threshold of 95% ANI was applied, which is commonly accepted. The resulting ANI matrix was used to construct a heatmap for visualization. The potential resistance and virulence factors were aligned against the Virulence Factors Database (VFDB) full dataset (setB) using BLASTp with an *E*-value cutoff of <10^−6^, identity >50%, and coverage >60% (Liu et al., 2022). The identification of antimicrobial resistance genes was performed using AMRFinderPlus (Feldgarden et al., 2021).

### 2.3 Comparative genomics, pan-genome analysis and phylogenetic analysis

For comparative genomic analysis with *Bacillus licheniformis*, a total of 498 *B. licheniformis* genome sequences (October 2025) were retrieved from the National Center for Biotechnology Information (NCBI). The dataset comprised 426 assembled genomes (Contig or Scaffold) and 72 complete reference genomes (chromosome or complete), the quality of assembly was assessed using CheckM v1.2.2 (Parks et al., 2015), resulting in 336 high-quality assemblies for downstream analysis, and 54 representative genomes were selected from these highly related *B. licheniformis* genomes (mash-distance <0.001). The pan-genome was determined for all isolates using Roary with the default setting (Page et al., 2015). The origin of transfers in DNA sequences of bacterial mobile genetic elements were searched with OriTFinder.^1^ Sequence comparisons between plasmids or chromosomes were performed using the BLAST Ring Image Generator (BRIG) (Alikhan et al., 2011). For 15 *Bacillus* spp. genomes, a genome-based phylogenetic tree was generated using TYGS. The evolutionary history was inferred by first determining the STs with the MLST (v2.23.0) program, followed by phylogenetic reconstruction with GrapeTree (v2.2). Core-genome MLST (cgMLST) analysis was performed using the chewBBACA (Silva et al., 2018) suite against a set of 1829 core genes. A maximum-likelihood phylogenetic tree, based on the concatenated alignment of pan genome was constructed using IQ-TREE (v 2.2.0.3) (Nunes et al., 2025). All bioinformatics analyses were performed within an Ubuntu Linux environment. Software dependencies and bioinformatics tools were installed and managed using Bioconda to ensure version control and reproducibility. All these phylogenetic trees were visualized using iTOL^2^ (Letunic and Bork, 2024).

### 2.4 Biofilm formation assay

Biofilm formation was assessed using a 96-well microtiter plate-based crystal violet staining method (Ben Abdallah et al., 2009). Overnight bacterial cultures were adjusted to OD_600_ = 0.5, diluted 1:50 in TSB with 1% glucose, and incubated at 37 °C for 36 h. Sterile medium served as negative control. After washing, adherent biofilms were fixed with methanol, stained with 0.1% crystal violet, and dissolved in 33% acetic acid. OD_570_ was measured. The cut-off value (ODc) was calculated as the mean OD_570_ of negative controls plus three times the standard deviation. Biofilm production was classified as: no producer (OD ≤ ODc), weak (ODc < OD ≤ 2 × ODc), moderate (2 × ODc < OD ≤ 4 × ODc), or strong (4 × ODc < 8 × OD) (Ben Abdallah et al., 2009).

### 2.5 Cytotoxic effect of strain LSDY01 on HEK293 cells

The cytotoxic effect of *B. licheniformis* LSDY01 on HEK293 cells was evaluated using the Cell Counting Kit-8 (CCK-8) assay (Ishiyama et al., 1996). Briefly, HEK293 cells were seeded into 96-well plates at a density of 1 × 10^4^ cells per well and cultured overnight to allow attachment. LSDY01 was grown to an optical density at 600 nm (OD_600_) of 0.5, diluted 1:50 in DMEM, and added to the cells at an approximate multiplicity of infection (MOI) of 100:1 for 24 h of co-culture. After gentle washing to remove non-adherent bacteria, CCK-8 reagent (10 μL per well) was added and the plates were incubated at 37 °C for 2 h. The absorbance at 450 nm (OD_450_) was then measured using a microplate reader (Thermo Fisher Scientific, USA). All values were corrected by subtracting the background absorbance of cell-free medium. Each experiment was performed in triplicate. Statistical significance was determined using a two-tailed independent samples t-test, with a *p*-value <0.05 considered statistically significant.

## 3 Results

### 3.1 Genomic features of strain LSDY01

**Figure 2:**
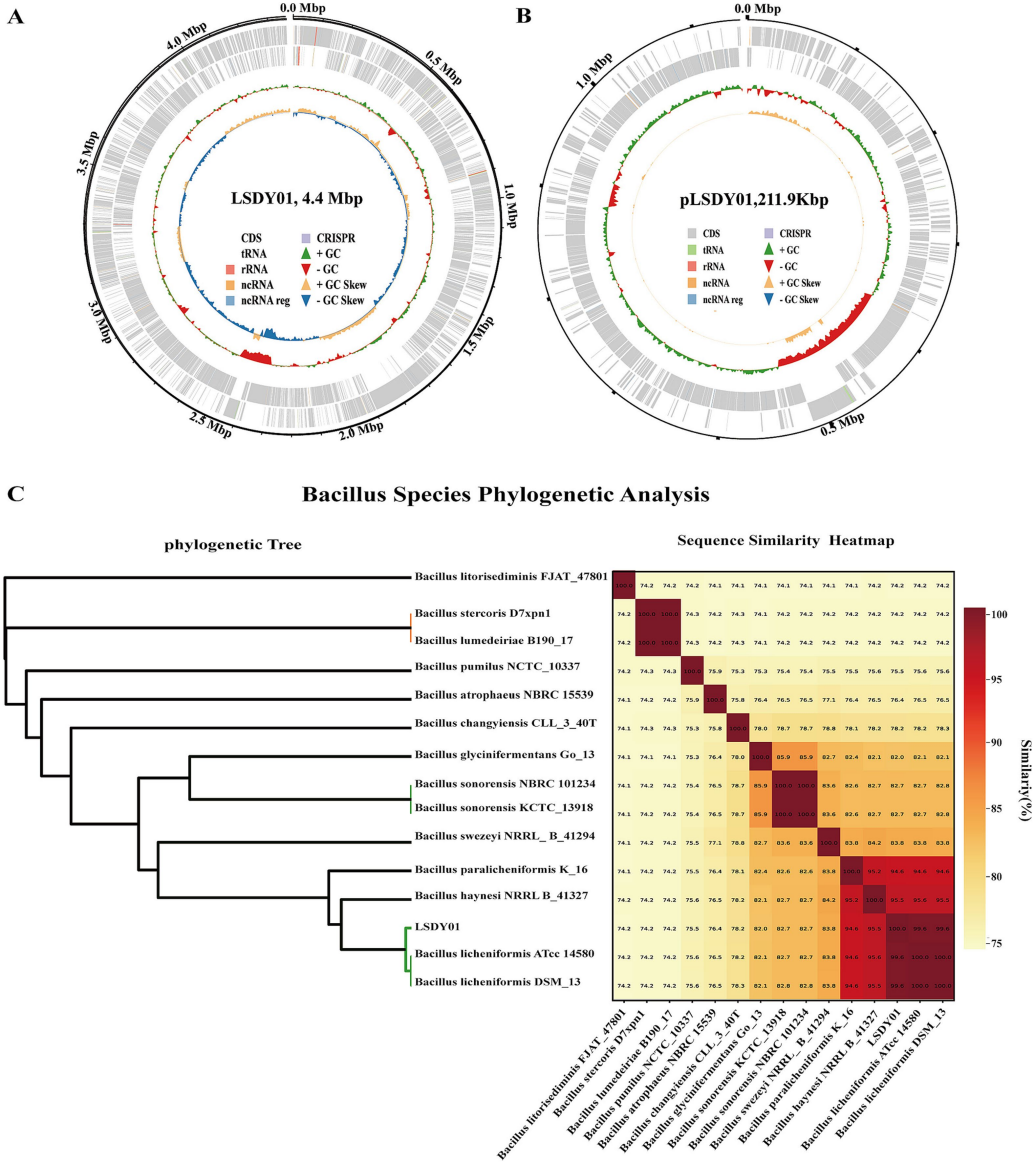
Genome visualization and annotation of strain LSDY01. **(A)** Chromosome; **(B)** plasmid. The element colour of each circle is indicated in the legend; **(C)** average Nucleotide Identity (ANI) heatmap illustrating genomic similarity among selected *Bacillus* strains. LSDY01 shows the highest ANI (99.6%) with *Bacillus licheniformis* ATCC 14580 and DSM 13, confirming its taxonomic assignment to this species. The color scale represents percentage similarity.

The *B. licheniformis* LSDY01(BioSample ID:SAMN53258325) genome contains a 4,363,059 bp chromosome with an average GC content of 45.63% and a 211,930 bp plasmid with an average GC content of 37.21%(Figures 2A,B). The general genomic features were given in Table 1. In total, 4,789 protein coding sequences (CDS) were predicted, with 4,490 in the chromosome and 299 in the plasmid. In silico analysis against the AMRFinderPlus database did not identify any acquired antimicrobial resistance genes on the chromosome or in the plasmid of strain LSDY01. A blastp analysis against the Virulence Factors Database (VFDB) and oriTfinder confirmed the presence of the *hlyIII, capA/capB, capD, clpC, tufA, groEL, lplA1, clpE, gnd, clpP, bpsC, lap, bslA/yuaB, dhb* gene cluster (*dhbABCDEF*), T4SS etc. in strain LSDY01 (see Table 2).

**Table 1:** Antibiotic resistance characteristics of *Bacillus licheniformis* LSDY01.

Antimicrobial class	Antimicrobial agent	Antibiotic minimum inhibitory concentration (μg/mL)	Result
Penicillins			
	Penicillin	≥0.25	R
	Ampicillin	**≥**0.5	R
Carbapenems			
	Imipenem	≤4	S
Glycopeptides			
	Vancomycin	≤4	S
Aminoglycosides			
	Amikacin	≤16	S
	Gentamicin	≤4	S
Macrolides			
	Erythromycin	≥8	R
Fluoroquinolones			
	Ciprofloxacin	≤1	S
	Levofloxacin	≤2	S
Lincosamides			
	Clindamycin	≥4	R
Folate pathway inhibitors			
	Trimethoprim-sulfamethoxazole	≥4/76	R
Phenicols			
	Chloramphenicol	≥32	R

**Table 2:** General features of *Bacillus licheniformis* LSDY01 genome.

Feature	Chromosome	Plasmid (pLSDY01)
Size (bp)	4,363,059	211,930
GC content (%)	45.63	37.21
CDS number	4,490	299
tRNAs number	89	1
tmRNAs number	1	0
ncRNAs number	97	1
5s rRNAs number	8	0
16s rRNAs number	2	0
23s rRNAs number	3	0

### 3.2 Phylogenetic analysis of strain LSDY01

The taxonomy of LSDY01 was analyzed using the Type (Strain) Genome Server (TYGS) tools based on a state-of-the-art genome-based taxonomy. The results showed that LSDY01 was most closely related to *B. licheniformis* ATCC 14580 or DSM_13 (Figure 2C). To better identify LSDY01 at the species level, a genome-based phylogenetic tree was constructed and ANI were calculated based on the genomes available from the TYGS analysis. An ANI value of 95–96% was used as a boundary for species delineation (Wambui et al., 2021). The results of ANI heatmap and phylogenetic relationship showed that LSDY01 had the highest ANI value (99.6%) and closest phylogenetic relationship to *B.licheniformis* ATCC 14580 or DSM_13 genomes. These results suggested that LSDY01 blongs to *B. licheniformis* based on the combined results of TYGS and ANI (Figure 2C).

**Figure 3:**
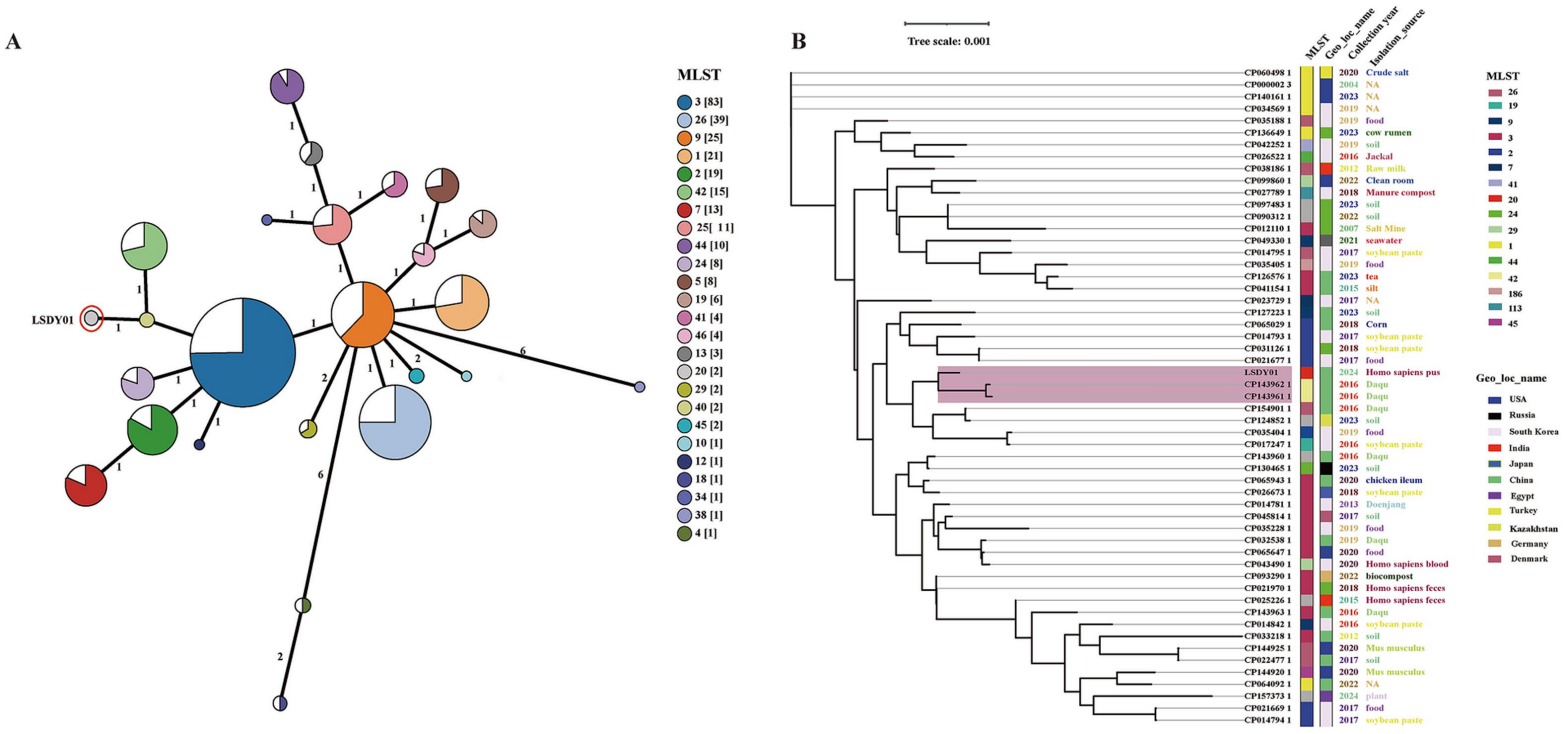
Molecular typing and genomic phylogeny of *Bacillus licheniformis* strain LSDY01. **(A)** In silico multi-locus sequence typing (MLST) reveals that LSDY01 belongs to the uncommon ST20, differing from the prevalent ST3 by a single allele. **(B)** cgMLST Phylogenetic analysis places LSDY01 in a clade with Daqu-derived strains CP143961.1 and CP143962.1 (bootstrap = 100), indicating recent divergence.

**Figure 4:**
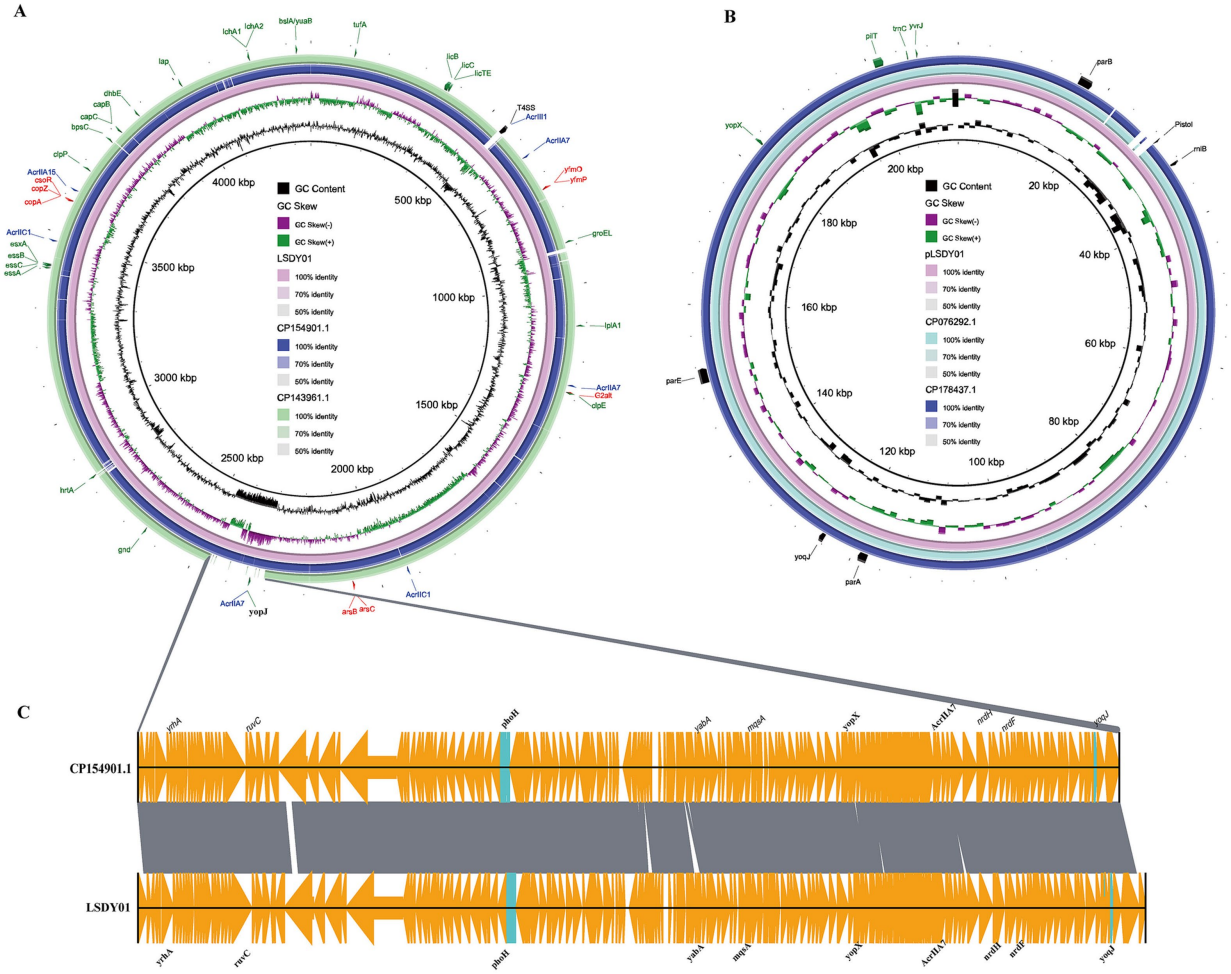
BRIG-based comparative genomic analysis of *Bacillus licheniformis* LSDY01. **(A)** Circular comparison of the LSDY01 chromosome with closely related strains CP143961.1 and CP143962.1. A unique ~157 kb genomic island (position ~2.3–2.5 Mb) was identified, characterized by significantly lower GC content (33.03%) and the co-localization of an anti-CRISPR gene (AcrIIA7) and a yoqS locus. **(B)** Plasmid pLSDY01 alignment with reference sequences CP076292.1 and CP178437.1, showing high identity (97.66%) and coverage (99%). The Pistol gene is uniquely present in pLSDY01. **(C)** Linear comparison of the ~157 kb genomic island with those of CP154901.1 and CP143961.1. The gray shading indicates regions of shared homology among different elements. The open reading frames are marked by colored arrows.

In silico multi-locus sequence typing revealed that LSDY01 belongs to ST20, which is uncommon among globally available *B. licheniformis* genomes, but it is just a single allele differences from the globally prevalent ST3 (Figure 3A). The closest evolutionary relatives of LSDY01 was CP143961.1 and CP143962.1 isolated from Daqu in China in 2016 (Figure 3B). The phylogenetic analysis showed that the branch connecting these sequences received maximum statistical support (bootstrap value = 100) and had a very short length (0.00031), indicating a recent and robust divergence. Comparative genomic analysis identified a ~157 kb horizontally acquired genomic island in strains LSDY01 and CP154901.1, characterized by a reduced GC content (33.03% vs. 45.63% in the core genome) (Figure 4A). This island harbors multiple virulence-associated genes, including those encoding a *yopX*, a ribonucleotide reductase complex (*nrdFHI*), the antitoxin MqsA, and the DNA repair endonuclease RuvC (Figure 4C). Notably, a type IV secretion system (T4SS) gene cluster was found on the chromosome of strain LSDY01. Comparative analysis against the PLSDB database revealed that plasmid pLSDY01 belongs to a highly conserved plasmid group, sharing near-complete sequence identity with CP076292.1 from Iranian soil and CP178437.1 from the gut of Chinese *Tenebrio molitor* larvae (97.66% identity, 99% coverage, *E*-value = 0.0), and carries no known antimicrobial resistance genes (Figure 4B).

Functional annotation of pLSDY01 identified key genetic elements, including a type II toxin-antitoxin system (*yvrJ-yoqJ*) for plasmid stability, a *yopX* potentially contributing to virulence, a type IV pilus retraction ATPase (*pilT*) possibly involved in twitching motility and natural competence, and the Pistol ribozyme with potential RNA-level regulatory functions. Together, these horizontally acquired elements, along with the strain-specific T4SS, likely enhance bacterial adaptability and pathogenic potential, contributing to the emergence of this lineage as an opportunistic pathogen.

### 3.3 Compare the pathogenic factors of *Bacillus licheniformis*

**Figure 5:**
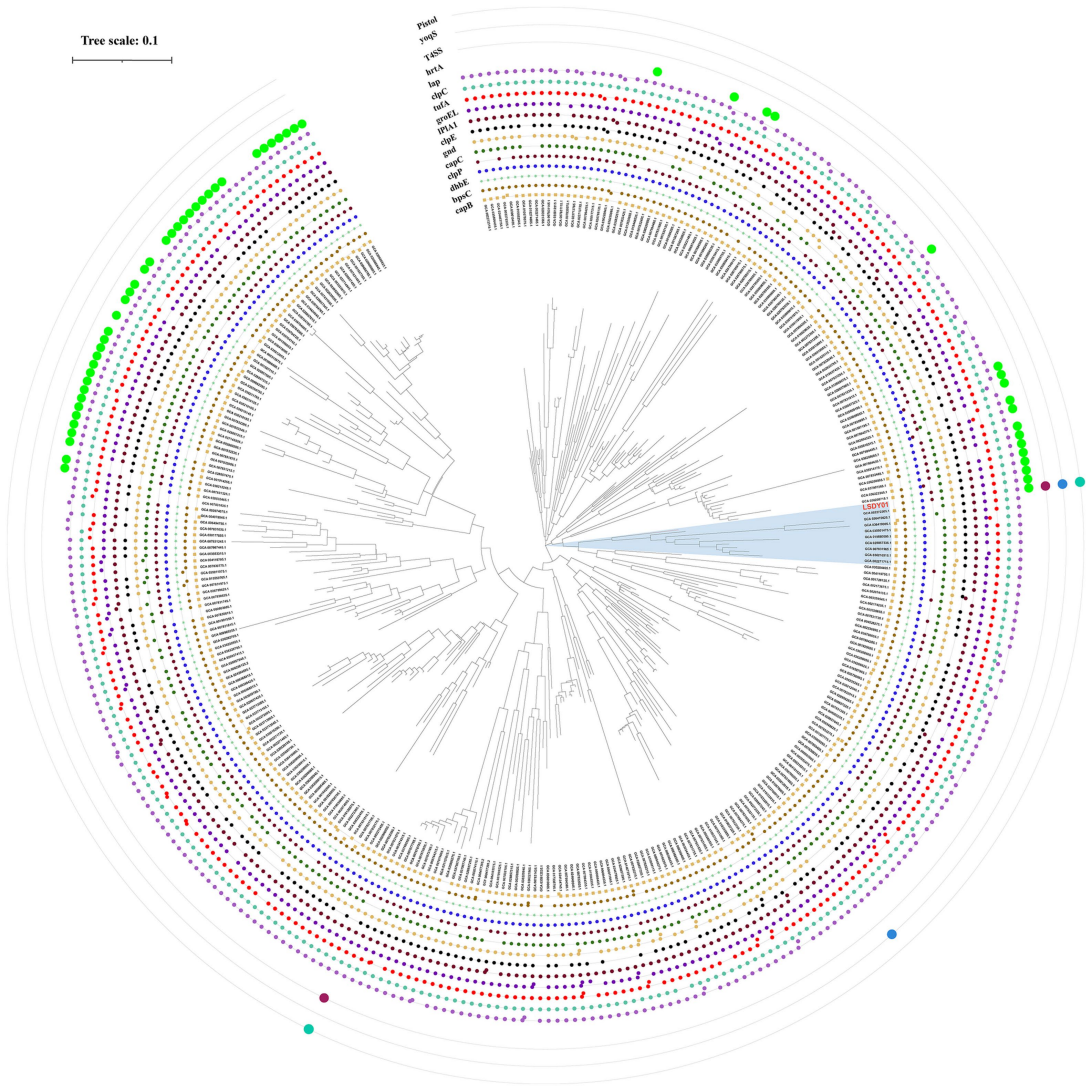
Phylogenetic distribution of virulence-associated genes in *Bacillus licheniformis* strains. Maximum-likelihood tree of 336 *Bacillus licheniformis* genomes. Notably, *yoqS, yoqJ*, and Pistol were uniquely identified in strain LSDY01, while *hrtA* was present only in a subset of strains, highlighting the distinct gene profile of LSDY01 compared to other *Bacillus licheniformis* isolates.

To assess the pathogenic potential of the LSDY01 strain, we profiled its virulence factors and identified 14 known or putative virulence genes against the Virulence Factors Database (VFDB) and oriTfinder (Li et al., 2018). These were classified into four functional categories: Adherence and Colonization (*hlyIII, capA/capB, capD, bpsC, lap*); Stress Survival and Host Adaptation (*clpC, clpE, clpP, groEL, tufA*); Nutritional/Metabolic Virulence (*dhb gene cluster, lplA1*); and Immune Modulation and Evasion (*gnd, bslA/yuaB*). Genomic comparison across 336 *B. licheniformis* strains confirmed that the aforementioned virulence-associated genes are widely conserved within the species. Meanwhile, other genetic elements showed strain-specific distribution: *yoqS*, *yoqJ*, and the *pistoL* gene were unique to plasmid pLSDY01; the T4SS locus was exclusive to strains LSDY01 and *B. licheniformis* GCA_003474315.1; and *hrtA* was present in only a limited subset of isolates (Figure 5).

### 3.4 Biofilm formation capacity of strain LSDY01

**Figure 6:**
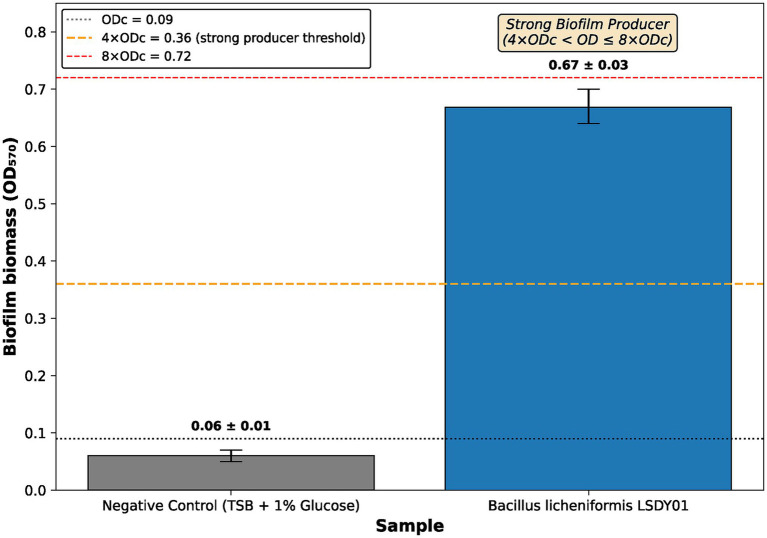
Strong biofilm formation by *Bacillus licheniformis* LSDY01. Biofilm biomass (OD_570_) was measured by crystal violet staining after 36 h at 37 °C in TSB with 1% glucose. LSDY01 (0.67 ± 0.03) was classified as a strong producer. Error bars: SD of quadruplicate wells.

The biofilm formation ability of *Bacillus licheniformis* strain LSDY01 was quantitatively assessed using the crystal violet staining method. The negative control (sterile TSB supplemented with 1% glucose) yielded a mean OD₅₇₀ of 0.06 ± 0.01, and the cut-off value (ODc) was calculated as 0.09 (mean + 3 × SD). As shown in Figure 6, strain LSDY01 exhibited a mean OD₅₇₀ of 0.67 ± 0.05 after 36 h of static incubation at 37 °C. Based on the classification criteria described, the OD₅₇₀ value of LSDY01 exceeded 4 × ODc (0.36), and fell within the range of 4 × ODc < OD ≤ 8 × ODc (0.36–0.72). Therefore, strain LSDY01 was classified as a strong biofilm producer. This result indicates that LSDY01 possesses a robust capacity for biofilm formation, which may contribute to its pathogenic potential by enhancing surface colonization and environmental persistence.

### 3.5 Cytotoxic effect of strain LSDY01 on HEK293 cells

**Figure 7:**
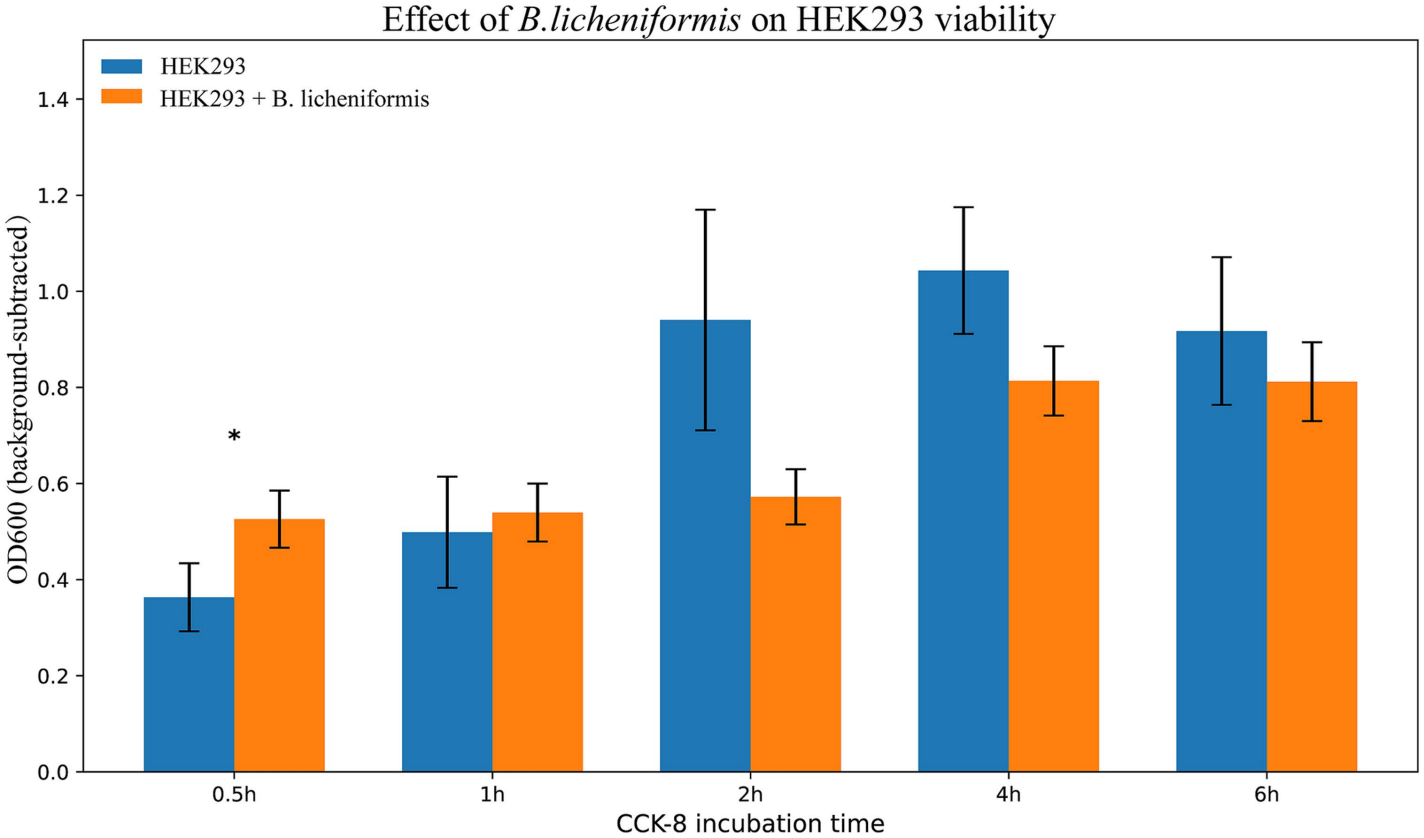
Effect of *Bacillus licheniformis* LSDY01 on HEK293 cell viability after 24 h co-culture. CCK-8 was added and absorbance (OD₄₅₀) was measured at the indicated time points. All values were normalized by subtracting the background signal of cell-free medium. Data are presented as mean ± SD (*n* = 3). Statistical significance was determined by independent samples *t*-test (two-tailed). At the 2 h, 4 h and 6 h readings, no significant differences were observed between the bacterial co-culture group and the control group (*p* > 0.05 for all).

The cytotoxic effect of LSDY01 on HEK293 cells was evaluated using a CCK-8 assay after 24 h of co-culture (Figure 7). After background subtraction, the bacterial co-culture group showed a 39% reduction in OD_450_ at the 2 h reading compared to the control group (0.572 ± 0.058 vs. 0.940 ± 0.230), although this difference did not reach statistical significance (*p* = 0.0545, two-tailed t-test). No significant differences were observed at any other time point (*p* > 0.05). These results indicate a trend toward reduced HEK293 cell viability following co-culture with LSDY01.

## 4 Discussion

The rising incidence of infections associated with *B. licheniformis*, a bacterium historically considered safe, underscores the need to reassess its pathogenic potential. In this study, we conducted a comprehensive genomic analysis of *B. licheniformis* strain LSDY01, isolated from a recalcitrant human skin infection. We identified a unique genetic architecture that may collectively contributes to host adaptation, persistence, and virulence. These findings challenge the conventional view of *B. licheniformis* infections as purely opportunistic, suggesting instead the emergence of genetically adept, highly adaptive lineages—a phenomenon consistent with evolutionary patterns observed in other opportunistic pathogens (Sheppard, 2022).

Genomic evidence suggests that the virulence of LSDY01 may be driven by a 211 stable novel plasmid (pLSDY01), along with two chromosomally located elements: a genomic island (GI) and a type IV secretion system (T4SS). Notably, the identification of the T4SS typically associated with pathogens (Paillard et al., 2025; Grohmann et al., 2017; Grohmann et al., 2018), the acquisition of a T4SS may enable LSDY01 to deliver effector proteins directly into host cells, subverting cellular functions and representing a substantial expansion of the virulence repertoire in this species. Functional annotation of plasmid pLSDY01 further identified a type IV pilus retraction ATPase (*pilT*). This gene may confer twitching motility, promoting surface colonization and biofilm formation, and is closely linked to natural competence (McCallum et al., 2019).

In support of this genomic prediction, phenotypic assessment revealed that LSDY01 is a strong biofilm producer (OD₅₇₀ = 0.67 ± 0.03), confirming its ability to form robust biofilms. This could significantly enhance the strain’s capacity for horizontal gene transfer, facilitating the acquisition of exogenous genetic material such as antibiotic resistance genes or novel virulence factors, thereby accelerating adaptive evolution and pathogenic diversification. The high conservation of backbone genes, including the toxin-antitoxin module *yvrJ-yoqJ*—a well-known mechanism for plasmid stability via post-segregational killing (Sarpong and Murphy, 2021), and the tRNA gene *trnC*—which may optimize translation and reduce metabolic burden (Zwanzig et al., 2019), suggests that core stability functions are preserved. In contrast, the unique acquisition of the Pistol ribozyme in the clinical isolate pLSDY01 points to potential novel regulation, as ribozymes can perform essential RNA processing and regulatory roles (Micura and Höbartner, 2020). Although genomic analysis predicted multiple virulence-associated determinants in LSDY01, functional assessment of its cytotoxic potential using a HEK293 cell co-culture model revealed only a non-significant trend toward reduced host cell viability (*p* = 0.0545 at 2 h of CCK-8 incubation). Nevertheless, a consistent numerical reduction was observed across multiple time points (2–6 h), suggesting a potential cytopathic effect.

The most notable genetic feature of LSDY01 is a ~157 kb genomic island with significantly reduced G + C content (33.03%), a hallmark of horizontal acquisition. The presence of the anti-CRISPR gene *AcrIIA7* adjacent to the *yopX* gene, which encodes a translocator essential for injecting virulence proteins into host cells during plague infection. We propose that *AcrIIA7* may have facilitated the acquisition of this and other mobile genetic elements by inhibiting the ancestral strain’s CRISPR-Cas system, thereby accelerating genomic plasticity and virulence evolution-a mechanism supported by studies on anti-CRISPR functions in other bacterial pathogens (Meeske et al., 2020).

Phylogenetic analysis classified LSDY01 within the ST20 lineage, differing from the widespread ST3 by only a single allele. This close relationship, together with the unique *AcrIIA7–yoqS* genomic island, indicates recent evolutionary divergence likely mediated by horizontal gene transfer. The isolation of its closest relatives from Daqu, a traditional fermentation starter, emphasizes the role of environmental and food-related niches as potential reservoirs for genetic exchange and emergence of pathogenic variants, corroborating recent insights into fermented-food microbiomes as platforms for gene exchange (Castañeda-Barba et al., 2024).

## 5 Conclusion and future directions

In summary, our genomic characterization and phenotypic validation suggests that *B. licheniformis* LSDY01 may represent a clinically relevant pathogen equipped with a sophisticated genetic toolkit and a strong biofilm-forming capacity. Moving beyond a mere gene inventory, we propose an integrated model wherein plasmid stability mechanisms, a horizontally acquired genomic island featuring an anti-CRISPR core, and dispersed virulence factors could act synergistically to promote infection. However, functional assessment of its cytotoxic potential using a HEK293 cell co-culture model revealed only a non-significant trend toward reduced host cell viability (*p* = 0.0545 at 2 h of CCK-8 incubation), suggesting that the strain may not exert a strong direct cytotoxic effect on this cell line under the tested conditions. This work underscores the importance of continuously re-evaluating the safety status of specific microorganisms as genomic and clinical evidence accumulates. A primary limitation of this study is its reliance primarily on *in silico* analyses with limited functional validation; therefore, further experimental validation of the proposed model remains for future investigation. Collectively, the genomic and phenotypic evidence indicates that *B. licheniformis* LSDY01 could represent an emerging clinical pathogen whose virulence might stem from synergistic interactions among plasmid stabilization systems, a horizontally acquired genomic island, and an array of chromosomal virulence determinants, as well as its intrinsic ability to form strong biofilms. These findings reinforce the necessity for an ongoing, evidence-based re-assessment of the pathogenic potential of microorganisms often considered non-pathogenic or low-risk.

## Data Availability

The datasets presented in this study can be found in online repositories. The names of the repository/repositories and accession number(s) can be found at: https://www.ncbi.nlm.nih.gov/genbank/, SAMN53258325.

## Footnotes

1
https://bioinfo-mml.sjtu.edu.cn/oriTfinder/

2
https://itol.embl.de/
